# A novel hybrid system for automatic detection of fish quality from eye and gill color characteristics using transfer learning technique

**DOI:** 10.1371/journal.pone.0284804

**Published:** 2023-04-25

**Authors:** İsmail Akgül, Volkan Kaya, Özge Zencir Tanır

**Affiliations:** 1 Department of Computer Engineering, Faculty of Engineering and Architecture, Erzincan Binali Yıldırım University, Erzincan, Türkiye; 2 Department of Biology, Faculty of Arts and Science, Erzincan Binali Yıldırım University, Erzincan, Türkiye; University of Wisconsin-Eau Claire, UNITED STATES

## Abstract

Fish remains popular among the body’s most essential nutrients, as it contains protein and polyunsaturated fatty acids. It is extremely important to choose the fish consumption according to the season and the freshness of the fish to be purchased. It is very difficult to distinguish between non-fresh fish and fresh fish mixed in the fish stalls. In addition to traditional methods used to determine meat freshness, significant success has been achieved in studies on fresh fish detection with artificial intelligence techniques. In this study, two different types of fish (anchovy and horse mackerel) used to determine fish freshness with convolutional neural networks, one of the artificial intelligence techniques. The images of fresh fish were taken, images of non-fresh fish were taken and two new datasets (Dataset1: Anchovy, Dataset2: Horse mackerel) were created. A novel hybrid model structure has been proposed to determine fish freshness using fish eye and gill regions on these two datasets. In the proposed model, Yolo-v5 and Inception-ResNet-v2 and Xception model structures are used through transfer learning. Whether the fish is fresh in both of the Yolo-v5 + Inception-ResNet-v2 (Dataset1: 97.67%, Dataset2: 96.0%) and Yolo-v5 + Xception (Dataset1: 88.00%, Dataset2: 94.67%) hybrid models created using these model structures has been successfully detected. Thanks to the model we have proposed, it will make an important contribution to the studies that will be conducted in the freshness studies of fish using different storage days and the estimation of fish size.

## 1. Introduction

Fish is a food that contributes to the protection and development of health in every period of life. Regular and adequate consumption of fish is recommended because it contains nutrients such as good quality protein, long-chain polyunsaturated n-3 fatty acids (n-3 LCPUFA), vitamin D and iodine.

In fish farming, fish morphometric feature measurement is a regular process on a daily basis in order to monitor the health and the growth of the fishes. The managers of fish farms need to measure manually the the body length and width (fish size), weight as well as color features of the eyes or the gills. Control and monitoring of fish freshness is very important in the food industry. The taste, health, quality and freshness of the fish are among the important factors. The changes during the storage of fish can be categorized based on apparent features including color, stiffness, odor, secretions, scales, skin, flesh, abdomen, eyes, and gills. These features can be used individually to assess the fish freshness [1].

Traditional methods used to determine fish freshness include sensory evaluations, chemical, physical measurements, and microbiological measurements are available. There exist various sensory (quality index method and torry scheme), physical (tissue analysis, torrymeter, electronic nose, and near-infrared reflection spectroscopy), and chemical and biochemical (measurement of total volatile nitrogen, trimethylamine, pH, and adenosine triphosphate) methods for this purpose [2]. However, there are some shortcomings in sensory analysis such as the high cost of the expert team, biased judgment due to fatigue and subjectivity, and the inability to use sensory analysis for continuous measurement. Chemical methods are objective and precise; however, they are normally used in a laboratory environment that damages fish and takes time. Microbiological enumeration methods are also destructive and require a very long time to culture bacteria and cannot provide test results quickly and effectively [3]. Therefore, it is necessary to develop new efficient, fast, cost-effective and non-destructive methods on fish fressness to overcome these problems.

Convolutional neural network (CNN) is one of the most popular deep learning methods used currently [4]. The most accepted and used CNN models in the literature are Yolo-v5, Inception-ResNet-v3 and Xception. Convolutional neural networks (CNN) shown outstanding success in image processing techniques, have achieved significant success in freshness fish studies. The recent development of convolutional neural networks (CNN) has led to great results in the field of identifying fish freshness. This study focuses on a novel method to address the progress of identifying fish freshness using a convolutional neural network.

An increasing number of studies include image analysis for different purposes in fish farming. In the literature, there are many studies based on image processing and by capturing the images of whole fish imaging [5–7], fillet imaging [8–23] and skin color imaging [24]. Most of previous researchers have focused on whole fish, fillet, and skin imaging and lately, few studies [1, 25–28] is available on application of machine vision technique on fish eyes and gills color changes during ice storage. In addition, many softwares have been developed in order to estimate the fish size [29–34].

The aim of this study is to evaluate the freshness of anchovy (*Engraulis encrasicolus*) and horse mackerel (*Trachurus trachurus*) based on its eyes and gills color changes by means of artificial intelligence methods. The effectiveness and applicability of the method is examined by a thorough investigation on features extracted from the eyes and gills images in different color spaces, and the classification of the icestorage duration using convolutional neural networks (CNN). The main contribution of this useful method is the use of non-destructive image preparation for sampling freshness. Another gain contribution of the verification method is the correct use of pixels belonging to fish eye and gill regions.

## 2. Materials and methods

### 2.1. Sample preparation ve system configuration

A group of 200 anchovy (*Engraulis encrasicolus*) and 200 horse mackerel (*Trachurus trachurus*) were obtained from local fish market, Erzincan, Turkey to experiment with our study [35]. All the fish were caught at the same time and in one lot by a seine net. The fish were transferred to the Laboratory of Erzincan Binali Yıldırım University in a polystyrene box with ice packs in less than 20 min. All procedures of the fish catch, storage and transfer were strictly monitored and controlled. The fish were first washed and then left to air dry for approximately 20 min. The samples were kept in the refrigerator with proper care. We know that the fish remains fresh in between 1–2 days. So we take the fresh sample in this time interval. After 1–2 days the color started to change. So in that time interval, we took the non-fresh sample. The duration of the assessment was about one week.

Fish images were taken without flash with a camera with 12M pixel size, 150dpi resolution, 4mm focal length and 1.9 F-stop. In addition, to detect and evaluate fish freshness in prepared samples, Yolo-v5, Inception-ResNet-v3 and Xception deep learning architectures were tested in a hybrid way by using Python programming language in Google Colaboratory [36] environment with NVIDIA Tesla K80 graphics processor.

### 2.2. Image pre-processing

**Fig 1:**
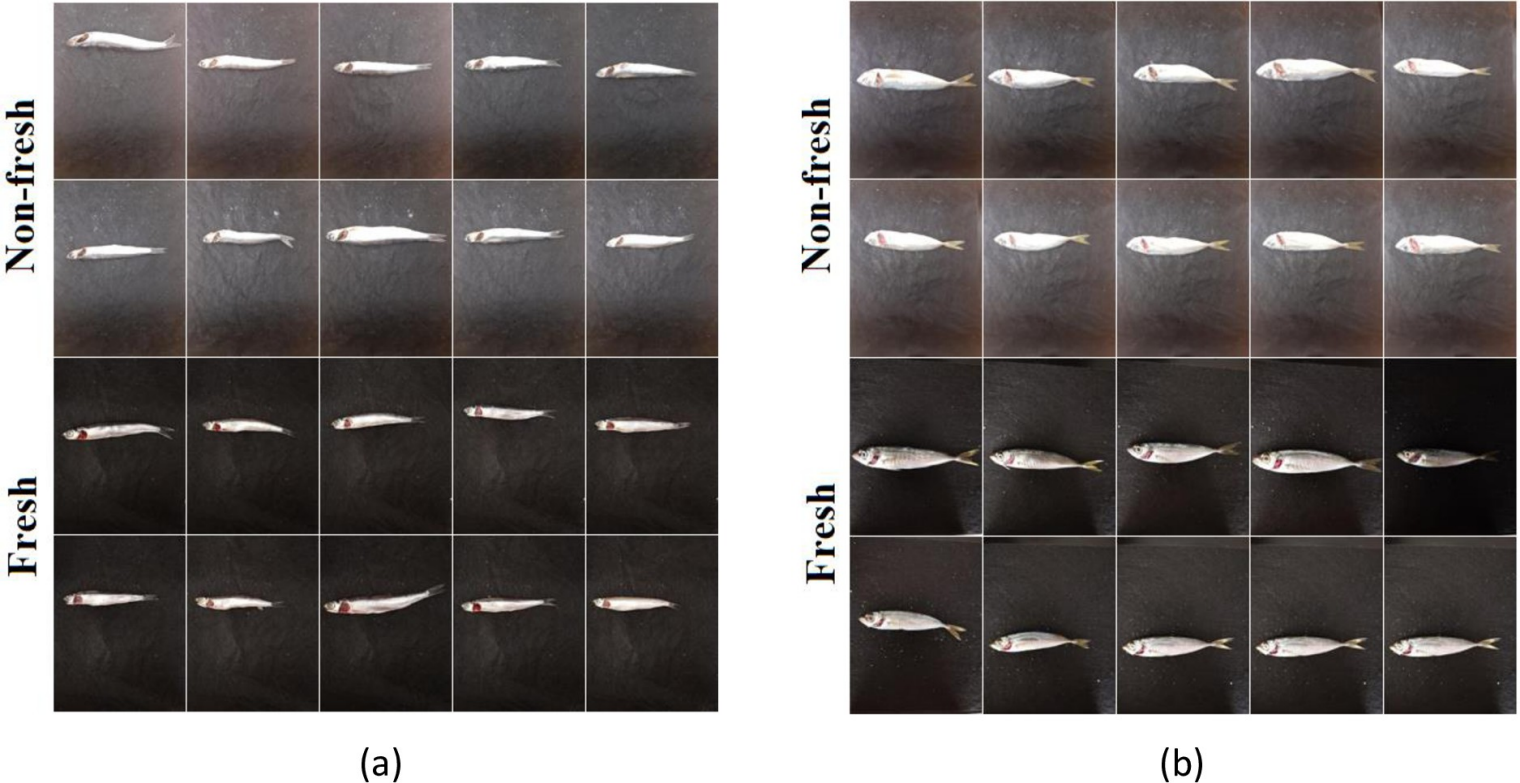
Fish freshness dataset sample images (a) anchovy (b) horse mackerel.

In this study, 200 anchovy (fresh and non-fresh) and 200 horse mackerel (fresh and non-fresh) fish images with 3000×4000×3 pixel size obtained during sample preparation were used. In order to increase the variety of fish images and the number of samples in this dataset, 10-fold data augmentation technique (rotation_range: %20, width_shift_range: %10, height_shift_range: %10, shear_range: %10, zoom_range: %10, horizontal_flip, vertical_flip ve fill_mode: ’nearest’) was applied and a total of 2000 anchovy and 2000 horse mackerel fish images were obtained. Since 2 types of fish were included in the study, the dataset was divided into 2 groups as anchovy and horse mackerel. Then, the fish images (anchovy-mackerel) in each group were randomly selected and reserved for Yolo-v5 model training (20%), Inception-ResNet-v2 and Xception model training (60%), and model performance testing (20%). Images of each fish species in the dataset are shown in Fig 1.

### 2.3. Proposed hybrid model structure

**Fig 2:**
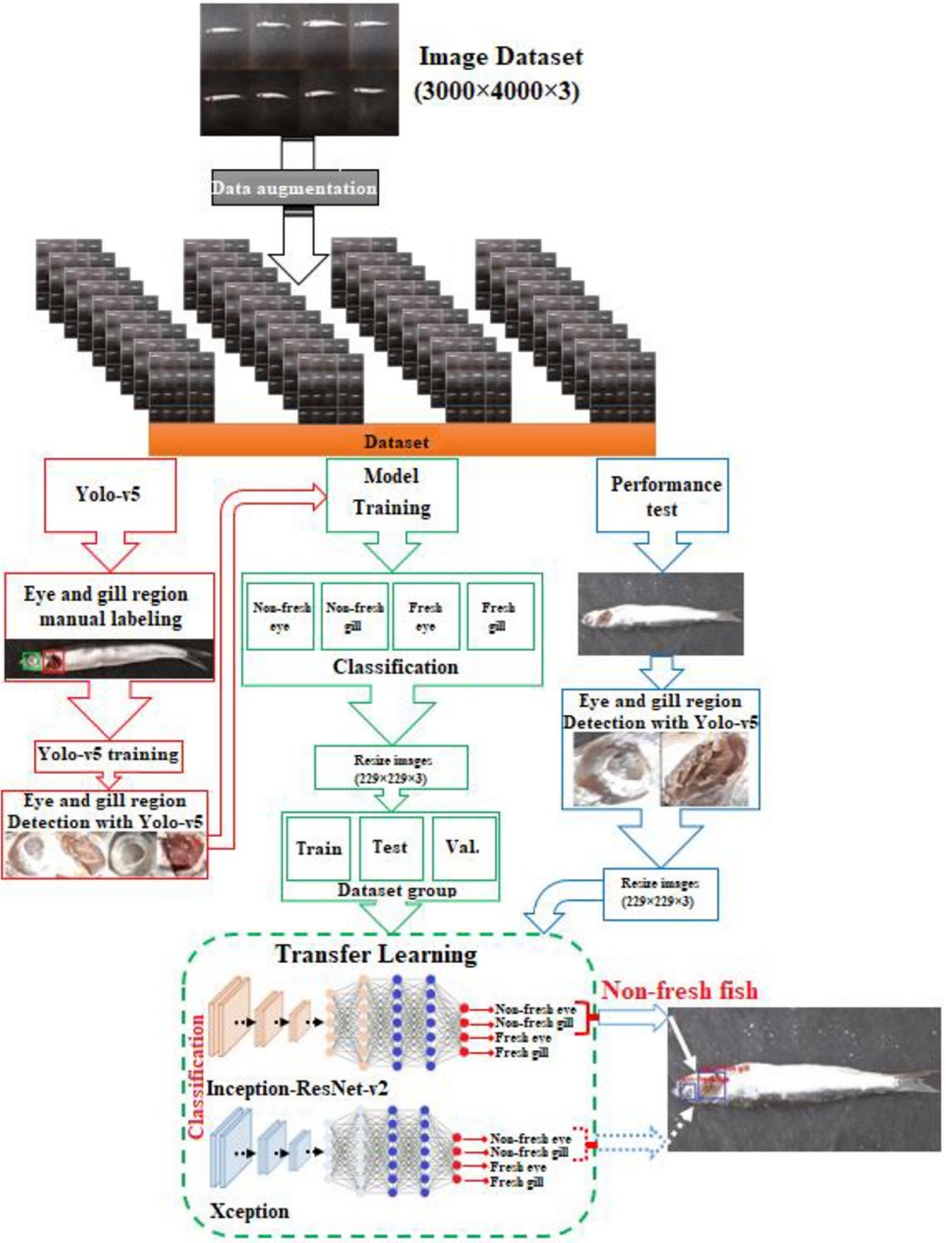
Proposed hybrid model structure.

In this study, a proposed hybrid model structure for fish freshness detection and classification is described. In this model structure, Yolo-v5 [37], Inception-ResNet-v2 [38] and Xception [39] model structures, which show superior performance in image detection and classification, were preferred and a new fish freshness detection method was developed. In order to create a novel model structure and extract features from our dataset, the previously trained Inception-ResNet-v2 and Xception model structures were used in a hybrid way with the Yolo-v5 model structure. The hybrid model structure given in Fig 2 is proposed to define whether the fish is fresh or non-fresh from the fish images.

In the proposed hybrid model structure (Fig 2), 200 anchovy (fresh and non-fresh) and 200 horse mackerel (fresh and non-fresh) fish species in RGB format with 3000×4000×3 pixel size prepared during the sample preparation process were first applied to the data augmentation technique. A total of 4000 images, 2000 of which belong to the fish species, were obtained. The fish images in the dataset were labeled as anchovy and horse mackerel and converted into 2 different datasets (Dataset1: Anchovy, Dataset2: Horse mackerel). The model was evaluated by giving Dataset1 and then Dataset2 separately to the proposed hybrid model structure. Then, the datasets of 2 different fish species were classified as fresh and non-fresh in order to be evaluated separately.

After classification, Dataset1: From 2000 fish images (fresh and non-fresh) in anchovy, 300 randomly fish images were reserved for Yolo-v5 training, 1400 fish images for classification model training, and 300 fish images for model performance testing.

In the proposed model, both fish eye and fish gill are taken into account to determine whether the fish is fresh or not. Therefore, fish eye and gill were manually selected in 300 fish images reserved for Yolo-v5 model training, and their region coordinates were labeled separately. The tagged fisheye and gill coordinates were then trained at 1700 epochs using the Yolo-v5 architecture. After the training, the eye and gill weights of the fish were obtained and the eye and gill regions of the fish were automatically determined using these weights. Eye and gill weights determined by Yolo-v5 were applied to 1400 fish image data group reserved for model training, and 1393 fish eye and 1400 fish gill region images were obtained.

These region images were collected under 4 different classes for model training as non-fresh eye, non-fresh gill, fresh eye and fresh gill. Thus, a Dataset1 of fresh and non-fresh eye and gill images was created and a total of 2793 data images were obtained. Then, the images in the dataset were resized at 229×229×3 pixels to be used in model (Inception-ResNet-v2 or Xception) trainings. After sizing, the dataset was divided into 3 data groups as training, testing and validation (Table 1). In order to perform the model training, transfer learning was applied by using the weights of the Inception-ResNet-v2 and Xception models. According to the training and test dataset, Inception-ResNet-v2 and Xception model trainings were carried out separately and the classification success rate was obtained. Then, the classification success rate of the models was verified with the validation dataset, which was not used in the training of the model networks.

**Table 1:** Image counts of the training, testing, and validation dataset.

Dataset	Training (%60)	Test (%20)	Validation (%20)	Total (%100)
Dataset1	1675	559	559	2793
Dataset2	1677	560	559	2796

In addition, the same operations were applied for Dataset2: Horse mackerel. Therefore, 300 random fish images from 2000 fish images (fresh and non-fresh) in Dataset2 are reserved for Yolo-v5 training, 1400 fish images for classification model training, and 300 fish images for model performance testing. Eye and gill weights determined with Yolo-v5 were applied to 1400 fish image data group reserved for model training, and 1398 fisheye and 1398 fish gill region images were obtained. Thus, a total of 2796 data images were obtained by creating a Dataset2 of fresh and non-fresh eye and gill images.

After creating Dataset1 and Dataset2, the number of images of the training, testing and validation datasets used in the evaluation process of the models are given in Table 1 in detail. In addition, the parameters given in Table 2 were used during the training process of the models.

**Table 2:** Models training parameters.

Parameters	Value
Epoch	15
Mini batch size	64
Activation function	Softmax
Optimization algorithm	Adamax

## 3. Results and discussion

In this study, fish eyes and gills were taken into account to determine whether the fish is fresh or not from the fish images. Therefore, using the Yolo-v5 architecture, firstly, the eye and gill regions of the fish were detected. The Yolo-v5 architecture was trained at 1700 epoch to successfully detect the eye and gill regions of the fish. The loss and average loss values obtained with the Yolo-v5 architecture as a result of the training are given in Table 3. Therefore, these low loss values obtained showed that Yolo-v5 architecture training was carried out successfully for both datasets.

**Table 3:** Yolo-v5 loss and avg loss values.

Dataset	Loss	Avg Loss
Dataset1	0.090	0.104
Dataset2	0.220	0.179

**Fig 3:**
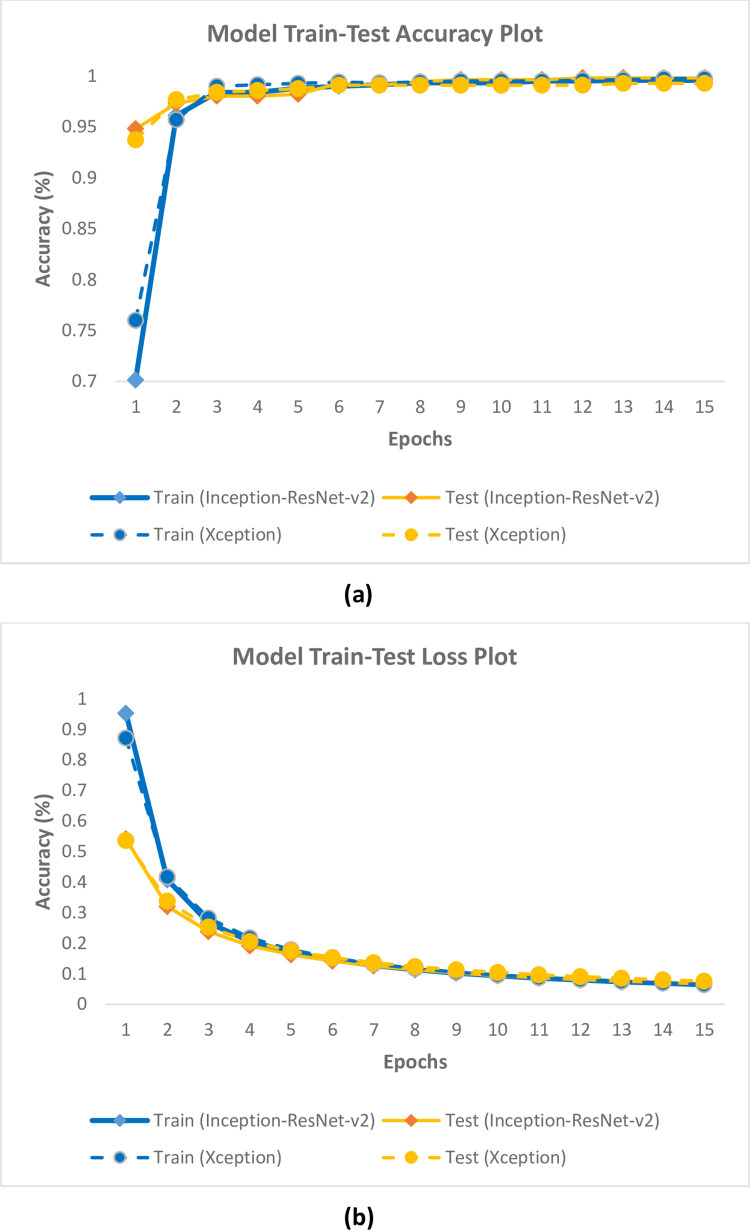
(a) Training-test accuracy, (b) Loss plots of models using Dataset1.

**Fig 4:**
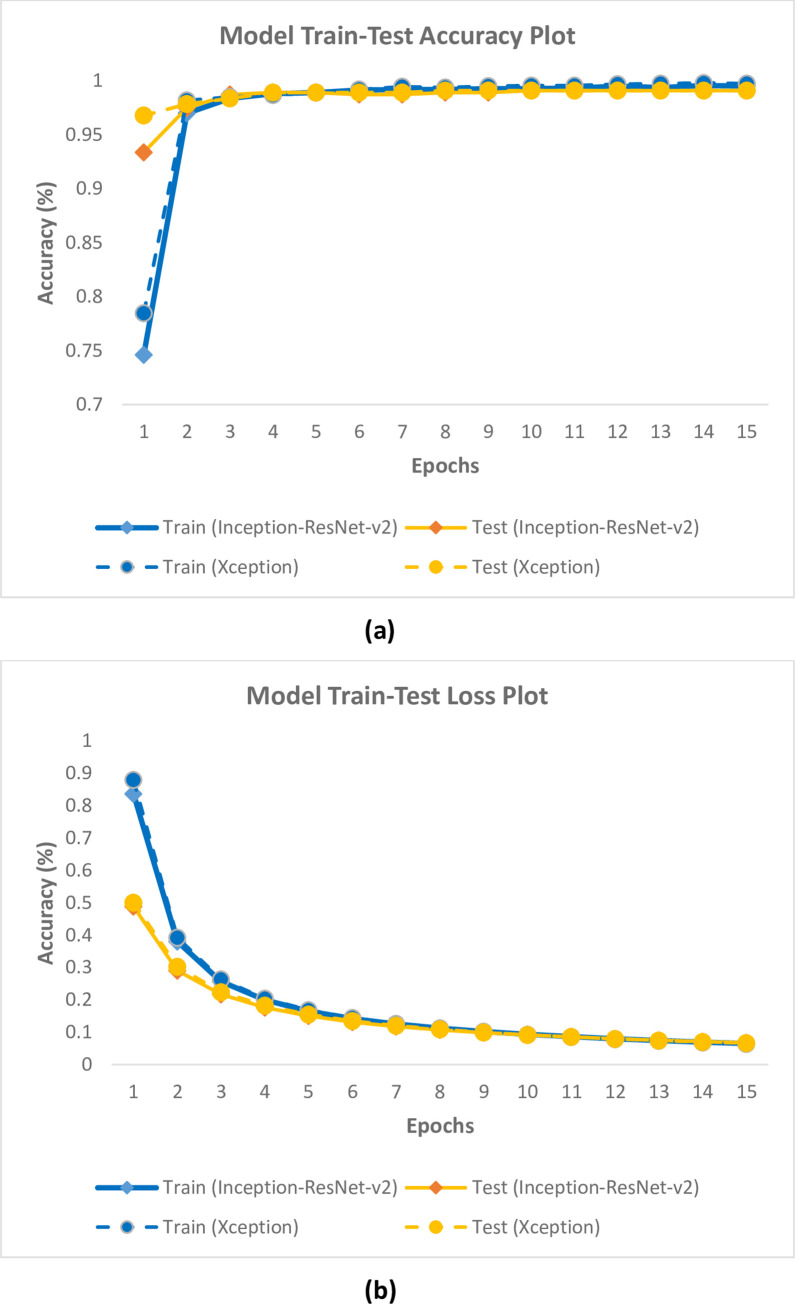
(a) Training-test accuracy, (b) Loss plots of models using Dataset2.

Dataset1 and Dataset2, consisting of fish eye (fresh and non-fresh) and fish gill (fresh and non-fresh) images obtained as a result of Yolo-v5 architecture training, were first divided into train, test and validation datasets to be evaluated separately. The training and test datasets were trained using classification model constructs (Inception-ResNet-v2 or Xception). As a result of the training, 4 classes were obtained as non-fresh eye, non-fresh gill, fresh eye and fresh gill. The results obtained using the training and test datasets are given in Table 4 in detail. In addition, the training-test accuracy graph obtained with Inception-ResNet-v2 and Xception models using Dataset1 is given in Fig 3A, and the training-test loss graph is given in Fig 3B. In addition, the training-test accuracy graph obtained with Inception-ResNet-v2 and Xception models using Dataset2 is given in Fig 4A, and the training-test loss graph is given in Fig 4B.

**Table 4:** Test results obtained from model structures using the training-test dataset.

Dataset	Model	Accuracy (%)		Loss	
		Train	Test	Train	Test
Dataset1	Inception-ResNet-v2	99.58	99.82	0.063	0.067
	Xception	99.76	99.28	0.064	0.076
Dataset2	Inception-ResNet-v2	99.52	99.11	0.065	0.067
	Xception	99.76	99.11	0.064	0.066

When the results in Table 4 and Fig 3 obtained using Dataset1 are examined, it is seen that the Inception-ResNet-v2 model has 99.58% training, 99.82% test success accuracy, and 0.063 training and 0.067 test loss values. It was seen that the Xception model reached 99.76% training, 99.28% test success accuracy and 0.064 training and 0.076 test loss values.

When the results in Table 4 and Fig 4 obtained using Dataset2 are examined, it is seen that the Inception-ResNet-v2 model has 99.52% training, 99.11% test success accuracy, and 0.065 training and 0.067 test loss values. On the other hand, it was seen that the Xception model reached 99.76% training, 99.11% test success accuracy and 0.064 training and 0.066 test loss values.

According to the results in Table 4, Figs 3 and 4, it was seen that the Inception-ResNet-v2 and Xception model trainings were successfully performed for both datasets used in the study. Inception-ResNet-v2 and Xception model trainings gave very close results. Therefore, the model trainings in fish freshness classification were considered successful.

In addition, to test the classification performance of model structures (Inception-ResNet-v2 or Xception), the classification success rates of the models were tested by using the validation dataset separated in Dataset1 and Dataset2.

**Fig 5:**
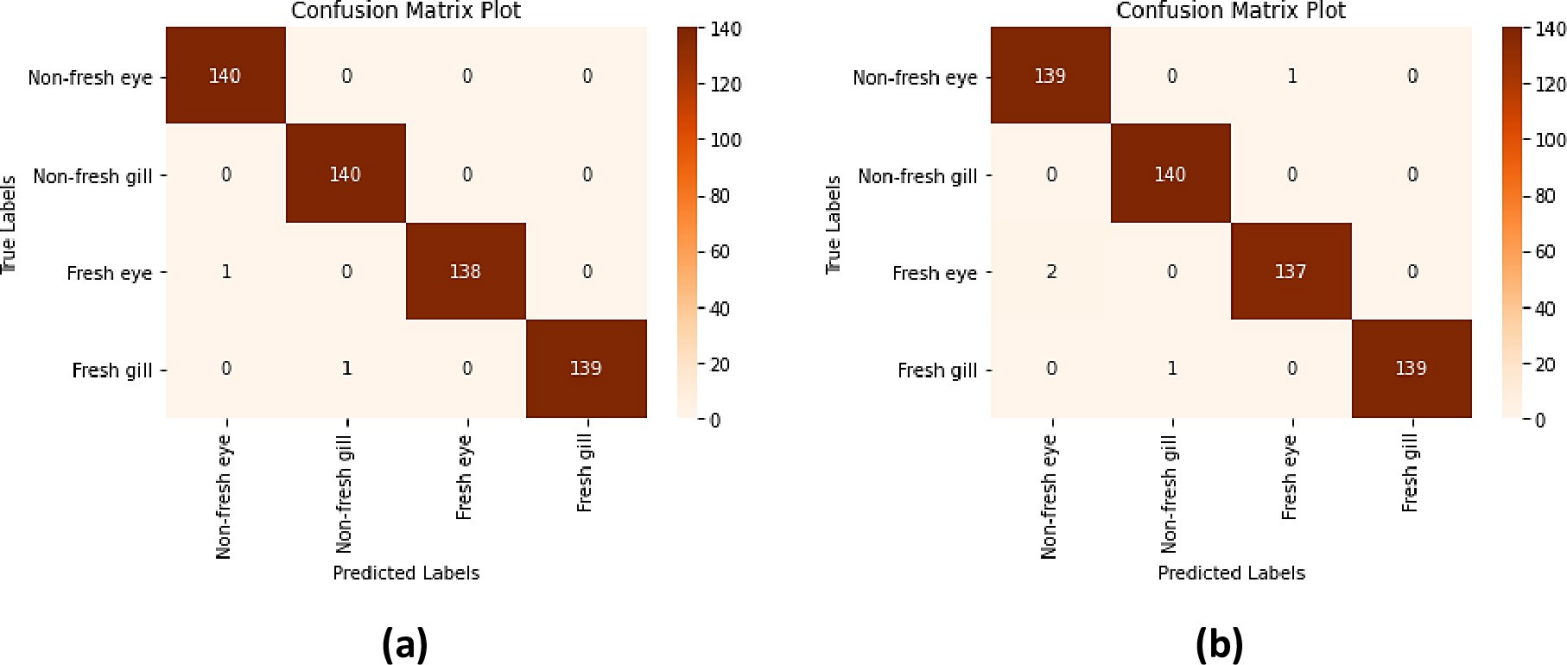
Confusion matrix results from (a) Inception-ResNet-v2, (b) Xception model structures using the validation dataset in Dataset1.

**Fig 6:**
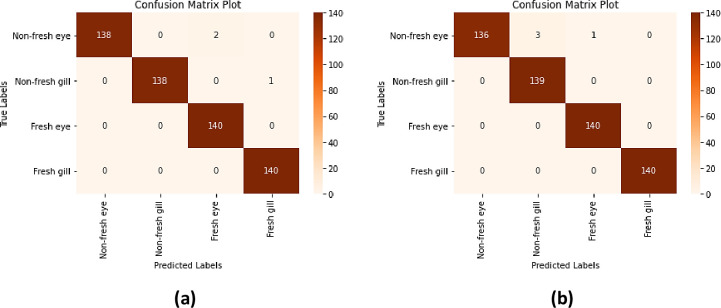
Confusion matrix results from (a) Inception-ResNet-v2, (b) Xception model structures using the validation dataset in Dataset2.

The success accuracy test results obtained using the validation dataset are given in detail in Table 5. In Figs 5 and 6, the confusion matrix of the results obtained using the validation data is given.

**Table 5:** Test results from model structures using the validation dataset.

Dataset	Model	Accuracy (%)	Loss
Dataset1	Inception-ResNet-v2	99.64	0.072
	Xception	99.28	0.076
Dataset2	Inception-ResNet-v2	99.46	0.074
	Xception	99.28	0.070

When the results in Table 5 and Fig 5 obtained using the validation dataset in Dataset1 are examined, it is seen that the Inception-ResNet-v2 model has 99.64% accuracy, 0.072 loss values, while the Xception model has 99.28% accuracy and 0.076 loss values.

When the results in Table 5 and Fig 6 obtained using the validation dataset in Dataset2 are examined, it is seen that the Inception-ResNet-v2 model has 99.46% accuracy, 0.074 loss value, while the Xception model has 99.28% accuracy and 0.070 loss value.

According to the results in Table 5, Figs 5 and 6, it has been seen that the results obtained with the validation dataset included in the two datasets used in the study make a successful classification similar to the training-test results of the Inception-ResNet-v2 and Xception models. In addition, it was concluded that the Inception-ResNet-v2 model classifies non-fresh eye, non-fresh gill, fresh eye and fresh gill images slightly better than the Xception model.

### 3.1. Performance evaluation

**Fig 7:**
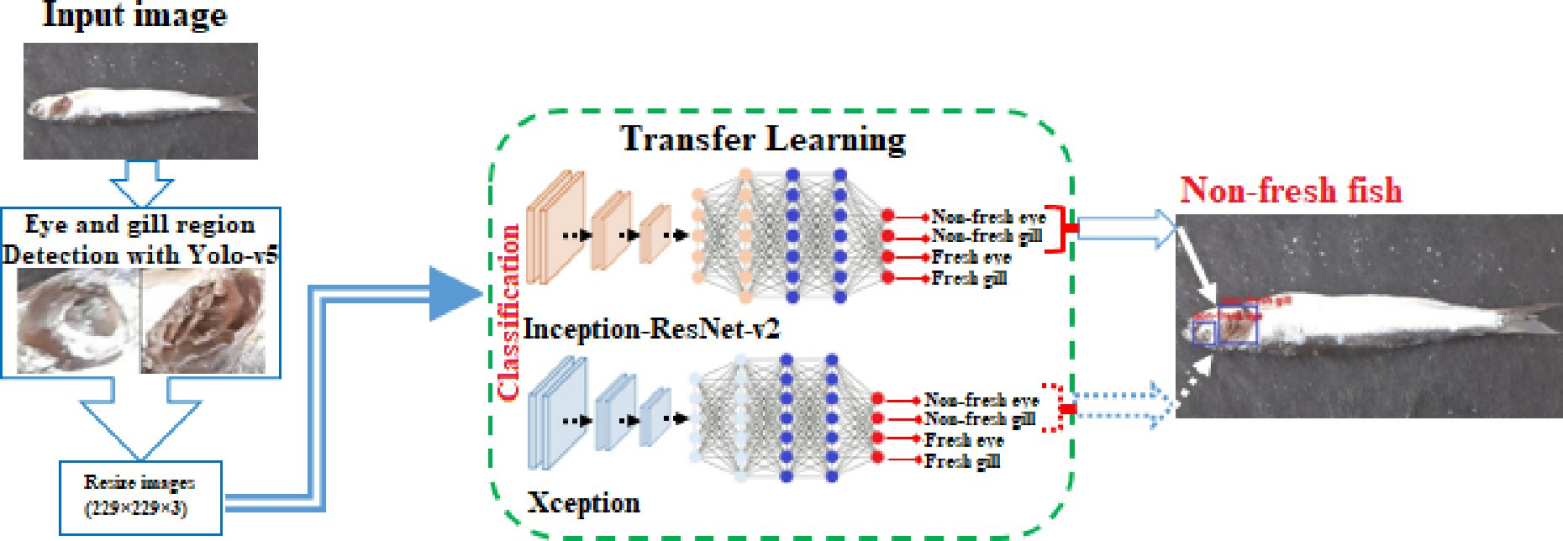
Proposed hybrid model structure performance diagram.

In the study, the proposed model structure was tested by using 300 anchovy and 300 horse mackerel fish images included in the dataset and reserved for performance testing. As a result of the test process, the coordinates of the eyes and gills on the fish image were determined and evaluated, and it was determined whether the fish given as the input image was fresh. In Fig 7, the model diagram of the performance test is given.

When the model diagram in Fig 7 is examined, the regions of fish eyes and gills were automatically determined and their coordinates were taken by using the Yolo-v5 model structure of the fish image that came as an input. Then, the obtained regions were resized to 229×229×3 pixels in order to be able to train the model. The eye and gill regions obtained as a result of this sizing were evaluated separately using Inception-ResNet-v2 and Xception model structures and classified as non-fresh eye, non-fresh gill, fresh eye and fresh gill. Since the method proposed focuses on both the eye and the gill of the fish, it will be able to decide whether the fish is fresh or not in environments that do not have a simple background. Therefore, the approach proposed will contribute to the fact that fish consumers can choose healthier fish in cases where they doubt whether the fish is fresh in any environment. As a result of the classification, the eye and gill coordinates on the fish image were taken and placed in a rectangular frame and the fish image was determined as fresh or not fresh according to the classification ratio. The success results of the fish images used in the performance test are given in Table 6 in detail.

**Table 6:** Performance test results of the proposed hybrid model.

Dataset	Model	Total Non-Fresh Fish	Non-Fresh Fish	Total Fresh Fish	Fresh Fish	Accuracy (%)
Dataset1	Yolo-v5 + Inception-ResNet-v2	150	143	150	150	97.67
	Yolo-v5 + Xception	150	115	150	149	88.00
Dataset2	Yolo-v5 + Inception-ResNet-v2	150	150	150	138	96.00
	Yolo-v5 + Xception	150	150	150	134	94.67

**Fig 8:**
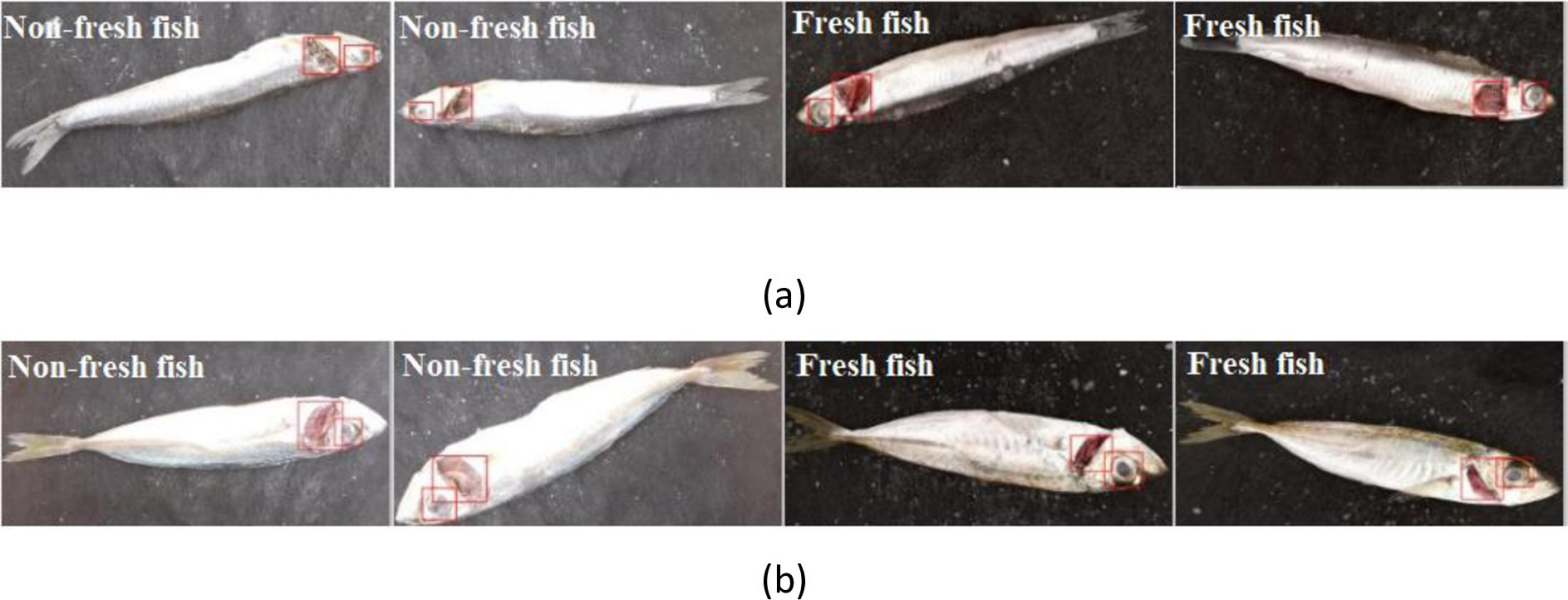
Sample images of the performance test (a) anchovy, (b) horse mackerel.

When the results given in Table 6 are examined, the rate of the proposed Yolo-v5 + Inception-ResNet-v2 model is 97.67% in Dataset1, 96.00% in Dataset2, 88.00% in Dataset1 of Yolo-v5 + Xception model, and %9 in Dataset2. It has been observed that it has a performance of 94.67 percent. According to these results, the Yolo-v5 + Inception-ResNet-v2 hybrid model performed better than the Yolo-v5 + Xception hybrid model in both datasets considered in fish freshness classification. In Fig 8, sample images of the performance test are presented.

In addition, the proposed hybrid model structures to determine fish freshness were compared with the methods available in the literature and are given in detail in Table 7.

**Table 7:** Comparison of proposed hybrid model constructs with existing methods to detect fish freshness.

References	Models	Dataset Images		Accuracy
		Fish Type	Number of Images	
[40]	ResNet-50	*Sparus aurata*	707	98%
	CNN	*Dicentrarchus labrax*		
	SVM	*Engraulis encrasicolus*		
		*Oncorhynchus mykiss*		
[41]	DSC-BE	*Chanos chanos*	4392	63.21%
		*Johnius trachycephalus*		
		*Nibea albiflora*		
		*Rastrelliger faughni*		
	RT	*Upeneus moluccensis*		
		*E*. *tetradactylum*		
	MB-BE	*Oreochromis mossambicus*		
		*Oreochromis niloticus*		
[42]	Neural network	*Oreochromis niloticus Cyprinus carpio*	150	94%
	Content-Based Image Retrieval	*O*. *mossambicus*		
[28]	VGG-16	Nile tilapia	4000	98%
	CNN-BiLSTM			
[43]	DCNN	Sardine	2127	99.5%
[27]	ANN	*Oncorhynchus mykiss*	20	96%
	SVM			
[44]	VGG-16	*Cyprinus carpio*	2464	98.21%
[45]	ABC-ANN	*Cyprinus carpio*	1344	93.01%
	SVM			
	K-NN			
	ANN			
**The proposed method**	**Yolo-v5 + Inception-ResNet-v2**	***Engraulis encrasicolus***	**5589**	**99.64**%
	**Yolo-v5 + Xception**	***Trachurus trachurus***		

## 4. Conclusions

In this study, a novel fish freshness detection model was developed to determine whether the fish to be purchased according to the season is fresh or not. In the developed model, the freshness of two different fish species (anchovy and horse mackerel) was determined. Since there is no ready-made dataset of the fish species discussed in the study, firstly, images of fresh fish caught with a single lot purse seine nets were taken. Afterwards, the fish were left under refrigeration conditions for a week to become stale and images of the stale fish were taken. Two different fish datasets (Dataset1: Anchovy, Dataset2: Horse mackerel) were created using the obtained fresh and stale fish images. The datasets of 2 different fish species were divided into fresh and non-fresh in order to be evaluated separately.

In order to extract a good feature from the datasets, the Inception-ResNet-v2 and Xception model structures, which were trained with transfer learning, were used in a hybrid way with the Yolo-v5 model structure. In the developed model, both fish eye and fish gill are taken into account to determine whether the fish is fresh or not.

It has been successfully determined whether the fish is fresh or not from the Yolo-v5 + Inception-ResNet-v2 (Dataset1: 97.67%, Dataset2: 96.00%) and Yolo-v5 + Xception (Dataset1: 88.00%, Dataset2: 94.67%) hybrid model structures developed using these model structures.

As a result, 2 different fish species (anchovy and horse mackerel) examined in the study showed superior success in detecting fish freshness by using the Yolo-v5 + Inception-ResNet-v2 and Yolo-v5 + Xception hybrid model structures. The findings obtained in this study will be helpful for fish consumers in determining the freshness of fish samples using artificial intelligence techniques. In addition, the applicability of the developed hybrid model structures will increase the importance of the study and will make a significant contribution to the freshness determination of different fish species to be made in this area. In future freshness studies on fish, a new classification dataset can be created with different artificial intelligence methods to determine fish freshness in order to show that the changes in gill and eye color of the fish can be more effective, taking into account the storage day (+3rd, 5th, 7th and 9th). Image processing and deep learning approaches that measure the morphological characteristics of fish can also be considered. In addition, it is thought that the hybrid models we have proposed for other marine fish species can be used for freshness detection.
